# Targeted Venetoclax Therapy in t(11;14) Multiple Myeloma: Real World Data From Seven Hungarian Centers

**DOI:** 10.3389/pore.2022.1610276

**Published:** 2022-02-28

**Authors:** Virág Réka Szita, Gábor Mikala, András Kozma, János Fábián, Apor Hardi, Hussain Alizadeh, Péter Rajnics, László Rejtő, Tamás Szendrei, László Váróczy, Zsolt Nagy, Árpád Illés, István Vályi-Nagy, Tamás Masszi, Gergely Varga

**Affiliations:** ^1^ Department of Internal Medicine and Haematology, Semmelweis University, Budapest, Hungary; ^2^ Department of Hematology and Stem Cell Transplantation, South Pest Central Hospital, National Institute for Haematology and Infectious Diseases, Budapest, Hungary; ^3^ Department of Molecular Genetics, South Pest Central Hospital, National Institute for Haematology and Infectious Diseases, Budapest, Hungary; ^4^ 1st Department of Internal Medicine, University of Pécs, Pécs, Hungary; ^5^ Department of Haematology, Teaching Hospital Mór Kaposi, Kaposvár, Hungary; ^6^ Faculty of Health Sciences, Institute of Diagnostics, University of Pécs, Pécs, Hungary; ^7^ Jósa András Teaching Hospital, Nyíregyháza, Hungary; ^8^ Teaching Hospital Markusovszky, Szombathely, Hungary; ^9^ Department of Haematology, Faculty of Medicine, Clinical Center, University of Debrecen, Debrecen, Hungary

**Keywords:** multiple myeloma, venetoclax, t(11;14), relapsed/refractory, plasma cell leukemia, amyloidosis

## Abstract

Despite the introduction of novel agents, multiple myeloma remains incurable for most patients, necessitating further therapeutic options. Venetoclax, a selective BCL-2 inhibitor, had shown promising results in patients with translocation t(11;14), but questions remain open about its optimal use. We have contacted all Hungarian haematology centers for their experience treating t(11;14) myeloma patients with venetoclax. 58 patients were reported. 37 received venetoclax in the relapsed/refractory setting with few or no other therapeutic options available. 21 patients started venetoclax as salvage after failing to achieve satisfactory response to first line therapy. In the relapsed/refractory setting objective response rate (ORR) was 94%, median progression-free survival (PFS) 10.0 months and median overall survival (OS) 14.6 months. In reinduction patients, ORR was 100%, median PFS and OS were not reached. Importantly, we found no adverse effect of high risk features such as deletion 17p or renal failure, in fact renal failure ameliorated in 42% of the cases, including three patients who became dialysis independent. Our study also reports the highest number of plasma cell leukemia cases successfully treated with venetoclax published in literature, with refractory plasma cell leukemia patients achieving a median PFS of 10.0 and a median OS of 12.2 months.

## Introduction

Despite advances in treatment in recent decades, multiple myeloma (MM) is still considered incurable, necessitating the development of newer therapeutic options. Triple class refractory patients—those refractory to an immunomodulatory drug (IMiD), a proteasome inhibitor (PI) and an anti-CD38 antibody—have a dismal outcome (1). Treatment options in this group consist of pomalidomide combinations, selinexor-dexamethasone, belantamab mafodotin, melflufen and BCMA chimeric antigen receptor (CAR) T-cells.

Patients refractory to induction treatment form another difficult to treat group. Proceeding to autologous stem cell transplantation (ASCT) is still considered standard of care, however outcomes with this approach are suboptimal: objective response rate (ORR) was 79% with 14.4 months progression-free survival (PFS) in the real life setting (2). The concept of a second line salvage treatment is controversial: in the Myeloma XI, a large randomized trial, deeper post-ASCT responses and PFS benefit has been demonstrated with second line PI salvage, whereas in a recent retrospective study by Jurczyszyn et al. found that immediate second-line ASCT was associated with better PFS (3,4).

Venetoclax is a selective B-cell lymphoma 2 (BCL-2) inhibitor now licensed in several different haematological malignancies, such as acute myeloid leukemia (AML) or chronic lymphocytic leukemia (CLL). The antiapoptotic BCL-2 protein belongs to the apoptosis regulating BCL-2 family. Upregulation of BCL-2 and other antiapoptotic BCL-2 family members such as MCL-1 or BCL-XL help cancer cells evade apoptosis by binding proapoptotic sensitisers and activators and may contribute to drug resistance (5,6). In certain myeloma cells that mainly rely on BCL-2 overexpression for the prevention of apoptosis - in the clinical setting, this correlates best with the presence of (11;14) translocation–venetoclax facilitates apoptosis induced by other drugs such as PIs (7).

The initial trials investigating venetoclax use in myeloma revealed evidence of single-agent antimyeloma activity in patients with relapsed/refractory (R/R) MM, predominantly in patients with t(11;14) (8). Further investigations temporarily stalled when interim analysis of the large phase 3 randomized double blind BELLINI trial comparing venetoclax-bortezomib-dexamethasone to bortezomib-dexamethasone in R/R myeloma revealed higher mortality in the treatment arm in spite of improved ORR (9,10). Several explanations, such as an increased risk for lethal infections have been proposed (9,11). Subgroup analysis revealed there was no such concern for t(11;14) patients, and the FDA approved further investigation in this subgroup. Subsequently promising results have been published in R/R patients (12,13), and in phase I and II trials evaluating use in combination with daratumumab (14), pomalidomide (15) and carfilzomib (16).

Translocation t(11;14) is found in approximately 15%–25% of all myeloma cases, historically conferring a prognosis not significantly different from the general myeloma population. Whereas the introduction of novel agents greatly improved prognosis in the overall population (17), this change may be less pronounced in t(11;14) myeloma, with some recent studies showing worse prognosis (18,19). Notably, the prevalence of t(11;14) is very high in two related plasma cell disorders: almost half of all patients with plasma cell leukemia or AL amyloidosis have this translocation (20,21), venetoclax use may therefore have a great impact in these patient groups. Case reports so far have described promising results in both AL amyloidosis (22–25) and PCL (26–31).

Pending the detailed results of randomized controlled trials, analysis of current clinical experience may provide valuable insight. Our report of all Hungarian myeloma patients treated with venetoclax-based therapy aims to contribute to this overview and help to answer some of the pressing concerns with the use of this medication.

## Materials and Methods

We contacted all Hungarian haematology centers, retrospectively surveying about their experience with venetoclax treatment in myeloma. Patients with more than one haematological malignancy, non-t(11;14) myeloma or those receiving venetoclax therapy for less than one complete cycle were excluded from our analysis.

We collected data about the patients’ cytogenetic makeup at diagnosis and relapse, international staging system (ISS) stage, prior lines of therapy, venetoclax dose, treatment duration and outcome, and adverse events associated with the drug.

Diagnosis, ISS staging and haematological response evaluation were performed according to the IMWG criteria (32). PFS and overall survival (OS) were calculated from the initiation of venetoclax therapy to the date of last medical contact or the date of progression and death, respectively. Refractoriness was defined based on the patient’s response to the last line containing the agent in question—if they failed to reach a PR or progressed on or within 2 months after finishing the protocol. Statistical analyses were carried out using the SPSS (version 26.0; SPSS, Chicago, IL, United States).

## Results

Seven Hungarian haematology centers participated in our study. 58 MM patients fitting our inclusion criteria were reported, having received venetoclax between August 2017 and August 2021.

Two different rationales behind venetoclax treatment were discernible: the majority, 37 patients received the drug in the R/R setting; whereas 21 patients underwent venetoclax therapy after suboptimal response to first line treatment, in preparation to ASCT.

### Patient Characteristics

Patient characteristics are detailed in Table 1. Relapsed patients were heavily pretreated with a median of 4 prior lines of therapy and were double (62%) or triple class (38%) refractory: the vast majority had previously received bortezomib, thalidomide and lenalidomide (95%, 86% and 89%, respectively), a significant portion was treated with later generation PIs (38% carfilzomib, 22% ixazomib), IMiDs (22% pomalidomide) or the anti CD38 antibody daratumumab (38%), treatment with which were stopped due to refractoriness in all cases. 59% of the patients had undergone ASCT. At the start of venetoclax therapy 57% had ISS stage 3 disease. High risk cytogenetic features were also very prevalent in this group: 32% of patients had 17p deletion and more than half (57%) had amp(1q).

**TABLE 1:** Patient characteristics.

Patient characteristics	Relapsed/refractory group (*n* = 37)	Reinduction group (*n* = 21)
Male gender	17 (46%)	9 (43%)
**Median age in years**		
At diagnosis	62 (32–86)	64 (50–91)
At the start of venetoclax therapy	69 (45–89)	65 (50–91)
Median time to venetoclax	4.7 years	2.6 months
**ISS stage at the start of venetoclax therapy**		
1	5 (14%)	11 (52%)
2	6 (16%)	1 (5%)
3	21 (57%)	9 (43%)
**Adverse prognostic factors**		
del(17p)	12 (32%)	6 (29%)
amp(1q21)	21 (57%)	8 (38%)
GFR <45 ml/min	11 (30%)	5 (24%)
**Prior therapy**		
Median lines of treatment	4 (1–12)	1 (1–2)
Single class refractory	0 (0%)	2 (10%)
Double class refractory	23 (62%)	5 (24%)
Triple class refractory	14 (38%)	0 (0%)

In contrast to the relapsed group where venetoclax therapy was started at a median of 4.7 years after diagnosis, the reinduction group was switched to a venetoclax-containing regimen after a median of 2.6 months. Prior treatment consisted of bortezomib (90%) and thalidomide (71%) for the most part, with a minority of the patients exposed to lenalidomide (14%). Del(17p) and add(1q21) were also found in this group (29% and 38% respectively).

### Combination Partners and Dosing

The majority of patients, 62% in the reinduction group and 65% in the R/R setting were treated with venetoclax in combination with bortezomib and dexamethasone. The remainder of the reinduction group, 38% received venetoclax as an add-on to the VTD therapy conventionally used in the first line, a combination unemployed in the heavily pretreated group. Other than with bortezomib and dexamethasone, the R/R patients received venetoclax either in monotherapy (22%) or added to carfilzomib-dexamethasone (KD, 14%).

Patients received individualised dosing of venetoclax, less in the reinduction cohort (mean 312 mg, range 150–400 mg) than in the R/R setting (mean 414 mg, range 200–800 mg). A significant majority in both patient groups (86% of all patients) took a concurrent antibiotic (i.e., clarithromycin, 73% of all patients) or antifungal (i.e., fluconazol, 12% of all patients) agent, the addition of which are known to increase venetoclax serum levels two-to fivefold. Relapsed/refractory patients were treated until progression, reinduction patients received a median of 3 cycles of venetoclax therapy before ASCT or observation.

### Efficacy

Considering the apparent difference in the clinical scenarios, we performed our analysis separately for the R/R and reinduction groups.

#### Relapsed/Refractory Setting

**FIGURE 1:**
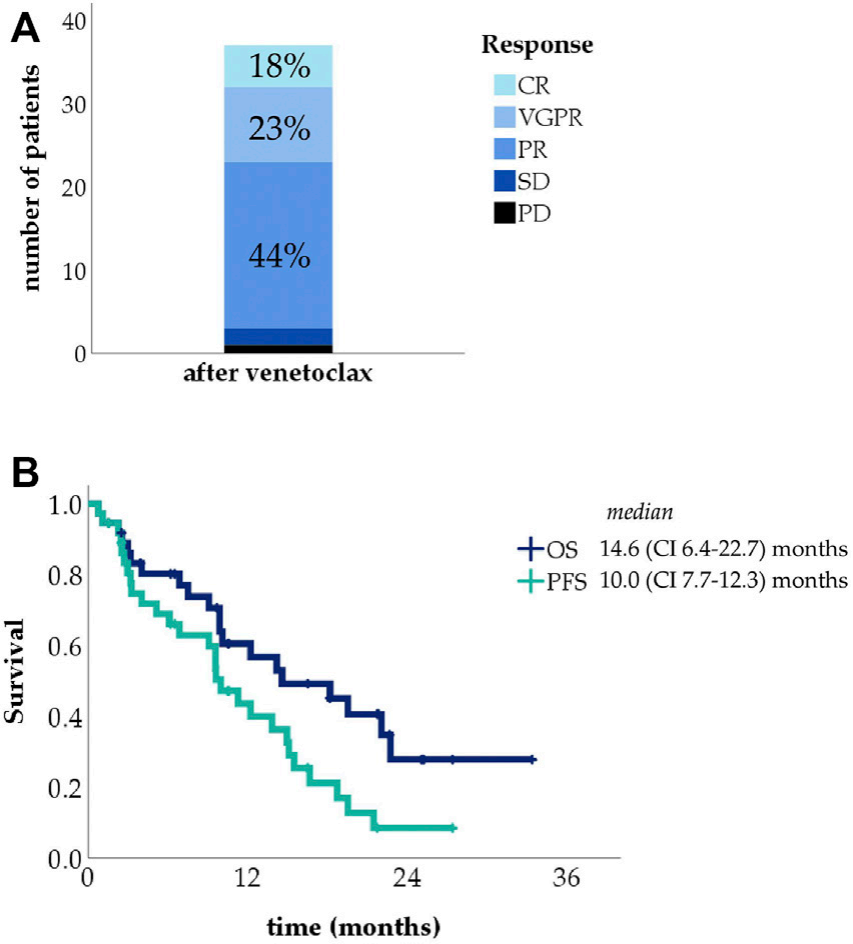
Treatment response rates in the relapsed/refractory group (CR, complete response, VGPR, very good partial response, PR, partial response, SD, stable disease, PD, progressive disease) **(A)**; Kaplan-Meier curves showing progression-free survival (PFS) and overall survival (OS) in the relapsed/refractory group **(B)**.

Given the refractoriness of this group, venetoclax therapy was associated with remarkably good results: all but three patients in the group had at least PR (ORR 92%), and 38% reached very good partial response (VGPR) or complete response (CR) (Figure 1A). Median PFS was 10.0 months, with median OS 14.6 months, as shown in Figure 1B.

#### Reinduction Setting

In the reinduction setting, venetoclax was started after suboptimal response to initial therapy: 13 patients had PR, 6 had stable disease (SD) and 2 progressive disease (PD), 5 patients were double class refractory. Patients received venetoclax for a median of 3 months and then proceeded to the planned ASCT or were switched to observation.

**FIGURE 2:**
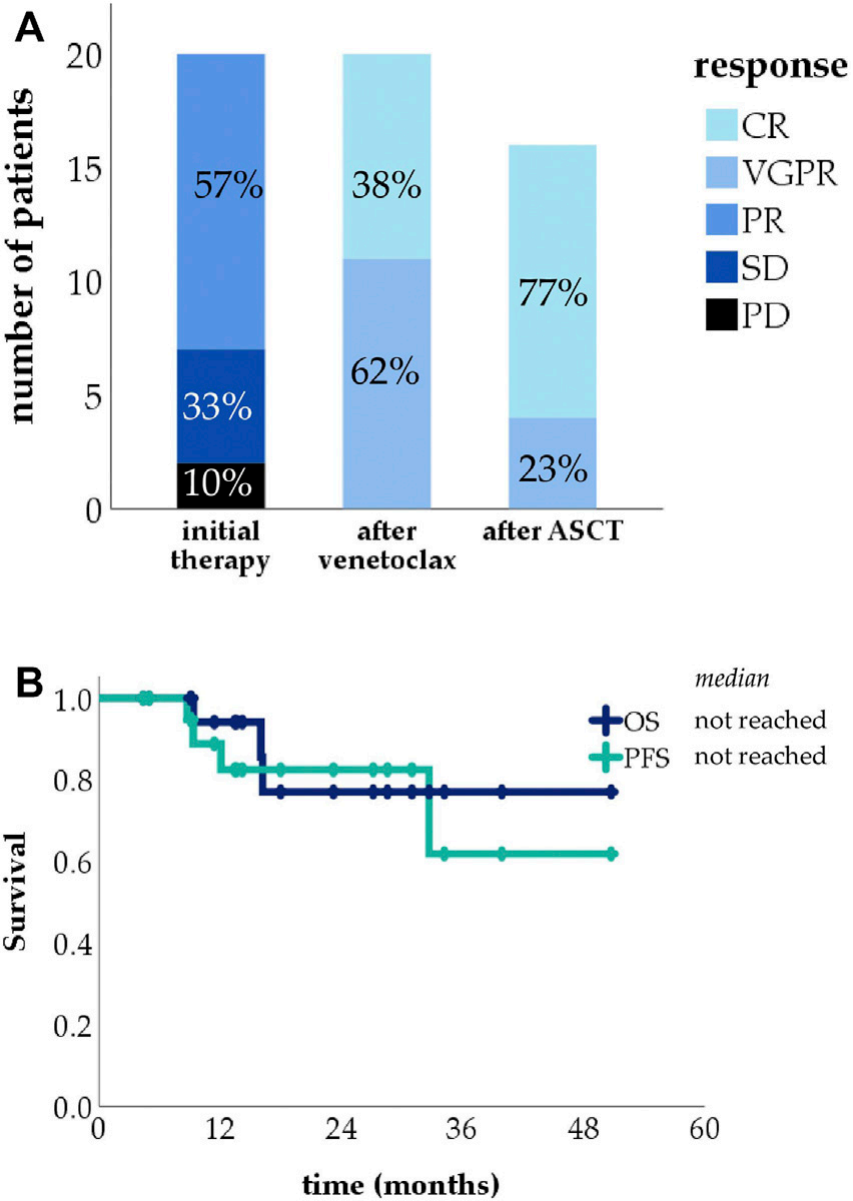
Treatment response rates in the reinduction group (CR, complete response, VGPR, very good partial response, PR, partial response, SD, stable disease, PD, progressive disease) **(A)**; Kaplan-Meier curve showing progression-free survival (PFS) and overall survival (OS) in the reinduction group **(B)**.

ORRs showed dramatic improvement after venetoclax use, with all patients reaching at least VGPR (Figure 2A) as judged from presentation as baseline. Of the 21 patients, 16 were eligible for ASCT, all of which were carried out. Median PFS and OS in this group were not reached (Figure 2B).

**FIGURE 3:**
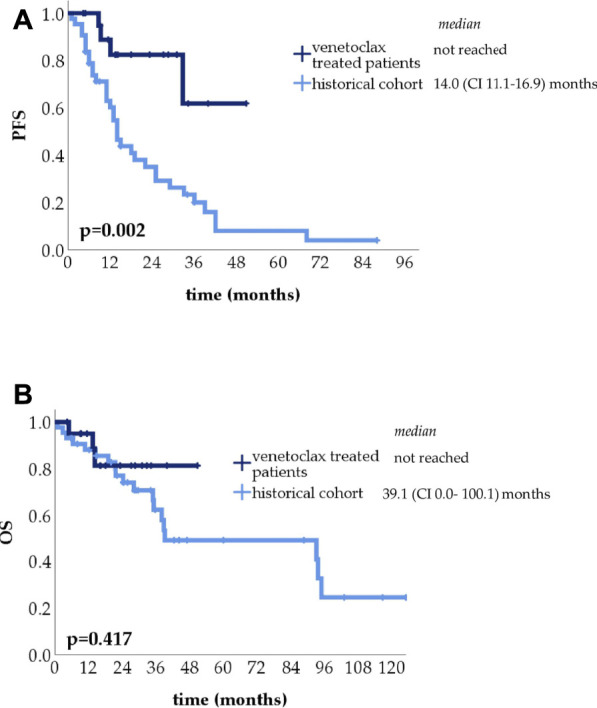
Progression-free survival (PFS) **(A)** and overall survival (OS) **(B)** comparison of the venetoclax-treated reinduction group and historical control.

To put these results into context we compared our results to a large dataset of t(11;14) myeloma cases treated without venetoclax presented earlier (33) from which we selected 43 who reached PR or less after an IMiD and/or PI based induction. 30 patients reached PR, 11 of whom proceeded to ASCT. Of the remaining 13 patients, 8 had SD and 5 PD, these received salvage treatment, 7 responded. The PFS of the venetoclax salvage group was significantly longer compared to the historical cohort (Figure 3A), without significant OS difference (Figure 3B).

### Prognostic Factors

**FIGURE 4:**
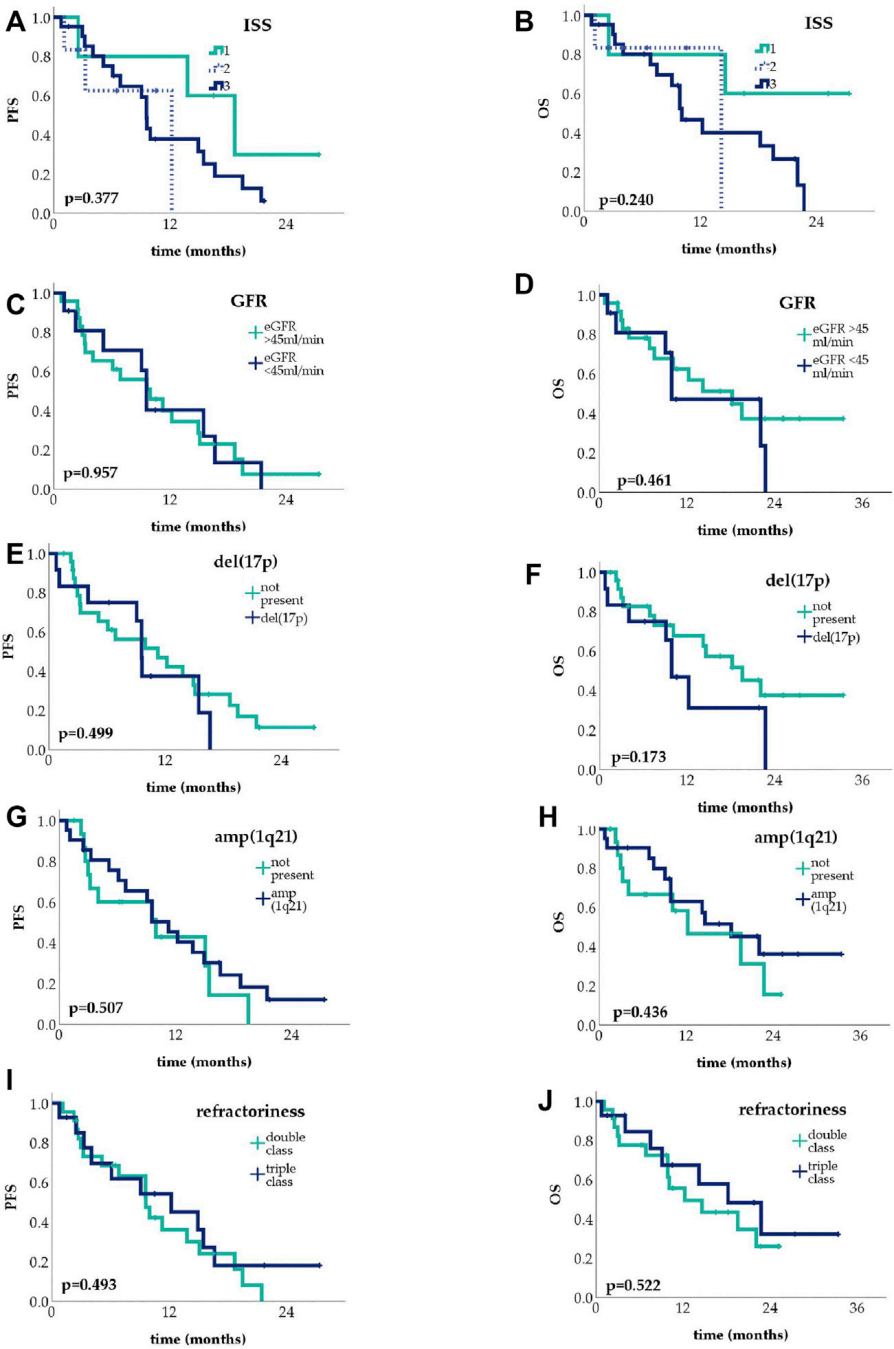
Kaplan-Meier curves showing progression-free survival (PFS) and overall survival (OS) in the relapsed/refractory setting, depending on International Staging System (ISS) stage **(A,B)**, kidney function **(C,D)**, del(17p) **(E,F)** and amp(1q21) **(G,H)** status, and refractoriness **(I,J)**.

We analyzed the effect of known adverse prognostic factors on PFS and OS. Since only four patients in the reinduction group had progressed, further analysis in this group is not yet possible. Subgroup analysis was performed in the R/R patient group (Figures 4A–H) subgroups’ PFS are given in the Supplementary Material.

No significant difference was found across the three ISS prognostic groups either in PFS (median 18.7, 12.2 and 9.6 months in ISS 1, 2 and 3, respectively; *p* = 0.377) or OS (median not reached, 14.2 and 10.1 months respectively; *p* = 0.240). There was no statistically significant difference between patients with or without kidney failure, defined as an eGFR <45 ml/min, either in PFS (median 9.6 vs. 10.0 months, *p* = 0.957) or in OS (median 9.9 vs. 18.2 months, *p* = 0.461). Similarly, in patients with or without del(17p), PFS (median 9.6 vs. 11.3 months, *p* = 0.499) and OS (median 9.9 vs. 19.5 months, *p* = 0.173) was not significantly different, just as the presence or absence of add(1q21) meant no significant difference in either PFS (median 11.3 vs. 10.0 months, *p* = 0.507) or OS (median 18.2 vs. 12.2 months, *p* = 0.436).

When comparing double-class and triple-class refractory patients, we found no significant differences, PFS were 9.6 and 12.2, OS 12.2 and 18.2 in double and triple refractory patients, respectively.

### Safety

Previously described adverse events associated with venetoclax use were tumour lysis syndrome, gastrointestinal complaints, cytopenias and infections. Just over half of the patients in our study experienced some kind of adverse event, more in the R/R group (60%) than in the reinduction group (38%), as detailed in Table 2.

**TABLE 2:** The incidence of adverse events in our study population.

Adverse event	Relapsed/refractory group (*n* = 37)	Reinduction group (*n* = 21)
Reported any adverse event	22 (59%)	8 (38%)
Gastrointestinal complaints (nausea/diarrhea)	6 (16%)	5 (24%)
Cytopenia	10 (27%)	1 (5%)
Infections	11 (30%)	2 (10%)
Tumorlysis syndrome	1 (3%)	0 (0%)
Acute myocardial infarction	4 (11%)	0 (0%)

The most common adverse events reported were infections, which were observed during the course of venetoclax treatment in 22% of the patients. Infections were reported in higher numbers in the relapsed setting than reinduction (30% vs. 10%, respectively) and had worse outcome: four of the infections in the former group proved lethal, two of which were COVID-19 related.

Gastrointestinal toxicity was observed in 19% of cases, with patients complaining of nausea or diarrhea. These side effects were for the most part mild and none necessitated discontinuation of therapy. Cytopenia involving one or more cell lines occurred in 19% of patients, affecting relapsed patients much more (27%) than reinduction ones (5%). Tumour lysis syndrome was reported in a single case only, in a patient with PCL.

It is noteworthy to mention that four patients in the relapsed group (11%) suffered ischemic coronary events during venetoclax use, one of which was fatal.

### Kidney Function

We examined the effect of kidney failure on outcomes in patients where data was available. 28% of the patients had impaired kidney function, defined as an eGFR <45 ml/min, at the start of venetoclax therapy, more in the relapsed (30%) than in the reinduction group (24%). As mentioned above, in the relapsed group there was no statistically significant difference between the PFS and OS of patients with or without kidney failure. After venetoclax therapy, we found clinically relevant improvement in kidney function in five of the patients (45%). Three patients required dialysis at the start of venetoclax therapy, importantly, dialysis could be stopped in all three of them.

Among patients with impaired kidney function, adverse events were much more prevalent: 83% of the patients with impaired kidney function vs. 37% of patients with eGFR >45 ml/min reported adverse events.

### Special Patient Groups

Considering the high prevalence of t(11;14) in plasma cell leukemia, as well as the limited treatment options available in this disorder, venetoclax use in this subgroup was an important aspect of our study.

Six of the reported patients had plasma cell leukemia, five in the relapsed, one in the reinduction group (Table 3). The reinduction patient and one patient in the R/R group had primary PCL (the latter progressing 4 years after the initial diagnosis), the four others in the relapsed group had secondary PCL detected at relapse.

**TABLE 3:** Number of plasma cell leukemia, extramedullary disease and amyloidosis cases in our study.

	All patients	Relapsed/refractory	Reinduction
Plasma cell leukemia	6	5	1
Extramedullary disease	4	3	1
Amyloidosis	5	1	4

Compared to the group medians, PCL patients were slightly younger (median 63 years old at diagnosis); had fewer prior lines of therapy (median 3.5) and a shorter time passed between the diagnosis and the initiation of venetoclax therapy (median 3.8 years). Half of the patients had del(17p) and an additional third add(1q).

ORRs to venetoclax therapy in PCL patients were remarkably good: all responded, 1 patient reached PR, 4 VGPR and 1 CR. Nevertheless, in the relapsed (secondary) group all patients progressed and passed away after a median PFS of 10.0 months, with a median OS of 12.2 months after the initiation of venetoclax therapy. The reinduction patient is still in CR, 31 months after the diagnosis.

Adverse events were very prevalent among PCL patients: 67% had infections during the course of venetoclax therapy, one of which proved fatal; and 83% had some degree of cytopenia.

## Discussion

Venetoclax-based therapy in translocation t(11;14) myeloma represents a unique targeted approach, however it is not yet approved by relevant authorities. In our real-world study, efficacy and safety of this approach was retrospectively studied on 58 patients who had individual off label usage approval and funding by Hungarian authorities.

In the relapsed/refractory myeloma setting, heavily pretreated double and triple class refractory patients had a 92% ORR to venetoclax treatment, giving patients a median PFS 10.0 and OS of 14.6 months after the start of venetoclax therapy. This compares favourably to the outcome of double class (PFS 5, OS 13 months) (34), and triple class refractory patients (PFS 3.4, OS 8.6 months) (1). This survival gain may benefit patients not only by itself, but also by bridging for more time-sensitive approaches such as transplantation or CAR T-cell therapy.

Considering the reinduction group, patients achieving suboptimal response after initial therapy usually do poorly, with ORR around 51% after the second-line therapy (3), while our cohort had a 100% ORR after venetoclax salvage, allowing eligible patients to proceed to ASCT and paving the way for long term remission. Median PFS and OS were not reached in this group, and longer follow-up is needed to allow comparison to standard therapies.

Analyzing different subgroups in our study, we have found that conventional prognostic factors such as ISS stage, kidney failure or del(17p) had no significant effect on PFS or OS. These results may indicate that patients with adverse prognostic factors especially benefit from venetoclax therapy. There is evidence that add(1q21) confers worse prognosis in patients treated with different regimens, including PIs, IMiDs and ASCT (35–39). Venetoclax-treated t(11;14) patients with gain/amp(1q21) may also fare worse, since this cytogenetic aberration is associated with elevated MCL-1 levels and offers myeloma cells an alternative antiapoptotic mechanism independent of venetoclax effect (40,41). The presence of gain/amp(1q21) was however not associated with worse prognosis in our study.

There is uncertainty concerning optimal venetoclax dosage in MM. Clinical trials utilized doses higher than that recommended in AML or CLL, e.g., 800 mg once daily (11). As detailed above, venetoclax administration in our study showed both interpatient and intercenter variance. In part due to gastrointestinal intolerance, and in part because of financial constraints associated with off label use of this drug, many physicians in our study combined venetoclax with moderate or strong CYP3A inhibitors, thus taking advantage of the necessary dose reduction. Most centers utilized clarithromycin, some voriconazol, according to local experience with these drugs. Adding clarithromycin to patients’ regimen, venetoclax dose must be reduced by 50% (42). Previous studies of venetoclax use in AML have shown that complying with the recommended 50% and 75% dose reductions kept venetoclax exposure comparable to the normal administration (43); and co-administration had no effect on long term outcomes (44). Patients were closely monitored during initial administration and in the centers where it was possible, serial serum level measurements were carried out. Despite lower venetoclax doses in our practice, venetoclax exposure in our patients was similar to that in the BELLINI study (11, 45).

Over half of the patients in our study encountered side effects or adverse events during treatment. The more serious of these were infections and cytopenias, events not uncommon in the general myeloma population. Their higher incidence in the relapsed group and disparately high rate in patients with kidney failure or plasma cell leukemia patients may be a consequence of the more aggressive disease itself, but nevertheless draws attention to the need for higher vigilance when using venetoclax in more vulnerable patients.

Although previous studies with venetoclax had listed acute coronary events among observed adverse events, an association had not been published (46). In our study we have found four cases of myocardial infarction. One patient was 56 years old, two of these patients were 64 years of age at the time of the event, whereas the patient we lost was 76 years old; three were male; two events were unexpected, with the other two patients having had severe pre-existing ischemic heart disease. Although this is a higher number than would be expected based on Hungarian epidemiological data of the general population (47), myeloma patients have been shown to have a higher risk of arterial thromboses and ischemic cardiac events. Studies have published the rate of ischemic cardiac events and myocardial infarction to be between 0.1% and 5.9% of patients, depending on drug combinations and patient characteristics (48-50). It is noteworthy that this rate was nevertheless very high in our studied population and further research is warranted to exclude a causative relationship with venetoclax-based therapy.

Clinical trials evaluating venetoclax excluded patients with impaired kidney function from participation. Previous pharmacokinetic studies have found that there is only minimal renal excretion of venetoclax (45), but little clinical experience has been reported (51). Our study found that although patients with impaired renal function had higher rates of adverse events, their PFS and OS after venetoclax treatment did not significantly differ from patients whose eGFR was above 45 ml/min. Importantly, venetoclax therapy was able to reverse myeloma-related renal failure in 42% of cases, overcoming dialysis dependence in three patients.

Our study had a disproportionately high number of plasma cell leukemia cases. Although case reports of successful venetoclax treatment of primary (26–28) and secondary (29, 30) plasma cell leukemia have been published, our study presents the highest number of cases available in literature so far. Plasma cell leukemia confers a prognosis significantly worse than MM itself: OS in secondary PCL is reported to be 1–4 months (21, 31), largely unchanged in recent years despite the introduction of novel agents. Among the relapsed patients, we found remarkably good PFS (10 months) and OS (12.2 months), demonstrating that this population may profit from venetoclax treatment tremendously.

## Conclusion

Our study shows that venetoclax-based treatment is a profitable option in both the R/R setting and also in reinduction patients not achieving optimal response with conventional therapy. Common adverse prognostic factors such as del(17p) appear to confer no disadvantage for patients with venetoclax treatment.

## Data Availability

The raw data supporting the conclusion of this article will be made available by the authors, without undue reservation.
