# Supplementary material for: Targeted Venetoclax Therapy in t(11;14) Multiple Myeloma: Real World Data From Seven Hungarian Centers
Source: Pathol Oncol Res. 2022 Feb 28;28:1610276. doi: 10.3389/pore.2022.1610276 (PMC8918485; doi:10.3389/pore.2022.1610276)
Supplement: Supplementary file 1 [file Table1.DOCX]

**Supplementary I.** Median progression-free and overall survival estimates in the relapsed/refractory group depending on International Staging System (ISS) stage, kidney function, del(17p) and amp(1q21) status, and refractoriness

|  | **Median progression-free survival (months)** | | | | **Median overall survival (months)** | | | |
| --- | --- | --- | --- | --- | --- | --- | --- | --- |
|  | Estimate | Standard Error | 95% Confidence Interval | | Estimate | Standard Error | 95% Confidence Interval | |
|  |  |  | Lower Bound | Upper Bound |  |  | Lower Bound | Upper Bound |
| **ISS stage** |  |  |  |  |  |  |  |  |
| 1 | 18.700 | 3.900 | 11.057 | 26.343 | not reached |  |  |  |
| 2 | 12.200 | 0.000 | . | . | 14.167 | 0.000 | . | . |
| 3 | 9.567 | 0.376 | 8.829 | 10.305 | 10.100 | 1.519 | 7.123 | 13.077 |
| **kidney function** |  |  |  |  |  |  |  |  |
| eGFR >45ml/min | 9.967 | 2.982 | 4.122 | 15.811 | 18.167 | 5.723 | 6.950 | 29.383 |
| eGFR <45ml/min | 9.567 | 0.385 | 8.813 | 10.321 | 9.867 | 4.569 | 0.911 | 18.822 |
| **del(17p)** |  |  |  |  |  |  |  |  |
| not present | 11.267 | 4.002 | 3.423 | 19.110 | 19.500 | 4.370 | 10.934 | 28.066 |
| del(17p) | 9.567 | 0.285 | 9.008 | 10.125 | 9.867 | 1.370 | 7.182 | 12.551 |
| **amp(1q21)** |  |  |  |  |  |  |  |  |
| not present | 9.967 | 4.728 | 0.700 | 19.233 | 12.200 | 5.126 | 2.153 | 22.247 |
| amp(1q21) | 11.267 | 1.730 | 7.876 | 14.658 | 18.167 | 4.419 | 9.505 | 26.828 |
| **refractoriness** |  |  |  |  |  |  |  |  |
| double class | 9.600 | 1.636 | 6.394 | 12.806 | 12.200 | 4.178 | 4.010 | 20.390 |
| triple class | 12.200 | 4.848 | 2.698 | 21.702 | 18.167 | 4.976 | 8.413 | 27.920 |
| ***Overall*** | *9.967* | *1.175* | *7.665* | *12.269* | *14.567* | *4.141* | *6.449* | *22.684* |
